# The Impact of Clinical Pharmacist Diabetes Education on Medication Adherence in Patients with Type 2 Diabetes Mellitus: An Interventional Study from Khartoum, Sudan

**DOI:** 10.3390/jpm14010074

**Published:** 2024-01-08

**Authors:** Safaa Badi, Sara Zainelabdein Suliman, Rayan Almahdi, Mohammed A. Aldomah, Mohamed ELsir Marzouq, Eiman Eltayeb M. Ibrahim, Musaab Ahmed, Mohamed H. Ahmed, Habab Khalid Elkheir, Mohamed Izham Mohamed Ibrahim

**Affiliations:** 1 Department of Clinical Pharmacy, Faculty of Pharmacy, Omdurman Islamic University, Khartoum 14415, Sudan; safi_alsaf@hotmail.com (S.Z.S.); rayan94.malik@gmail.com (R.A.); mohammedalshuraney@gmail.com (M.A.A.); mohamedelsir201317elsir@gmail.com (M.E.M.); eiman.eltayeb@oiu.edu.sd (E.E.M.I.); hababelkheir@smcreg.gov.sd (H.K.E.); 2 College of Medicine, Ajman University, Ajman P.O. Box 346, United Arab Emirates; m.omer@ajman.ac.ae; 3 Department of Medicine and HIV Metabolic Clinic, Milton Keynes University Hospital NHS Foundation Trust, Eaglestone, Milton Keynes MK6 5LD, UK; mohamed.hassan-ahmed@mkuh.nhs.uk; 4 Department of Geriatric Medicine, Milton Keynes University Hospital NHS Foundation Trust, Eaglestone, Milton Keynes MK6 5LD, UK; 5 Department of Clinical Pharmacy, University of Science and Technology, Khartoum 14411, Sudan; 6 Department of Clinical Pharmacy and Practice, College of Pharmacy, QU Health, Qatar University, Doha P.O. Box 2713, Qatar; mohamedizham@qu.edu.qa

**Keywords:** medications, adherence, diabetes, clinical pharmacists

## Abstract

Background: Continuous therapeutic care with good medication adherence is the cornerstone of management of all chronic diseases including diabetes. This study aimed to evaluate the impact of clinical pharmacist intervention on the medication adherence in individuals with type 2 diabetes (T2DM). Methods: This was a randomized, double-blind, controlled trial conducted at a diabetes clinic located at Omdurman Military Hospital, Sudan. Individuals with T2DM attending the diabetes clinic within 1 year were selected. The sample size was 364 participants (182 control and 182 interventional group). We used a pre-structured standardized questionnaire and checklist to collect the data. Data were analyzed by using the Statistical Package for the Social Sciences (SPSS) (version 28). Results: Majority, 76.4% (*n* = 278) were females, and they consisted of 80.8% (*n* = 147) of the interventional group and 72% of the controls. The mean age of the interventional group was 54.5 (±10) years; 31.9% (*n* = 58) of the interventional group had diabetes for 6–10 years, compared with 26.4% (*n* = 48) of the control group. Among the control group, the mean adherence score was 6.8 (±1.7) at baseline and it was 6.7 (±1.6) at the end of the study (*p* < 0.001), while in the interventional group, the mean adherence score was 6.8 (±1.7) at baseline and it was 7.4 (±1.5) at the end of the study (*p* < 0.001). Conclusion: Adherence score among the intervention group was increased significantly from baseline to the end of the study when compared to the control group.

## 1. Introduction

Diabetes mellitus is a well-recognized chronic metabolic disease and with poor control. It can lead to serious complications in the heart, kidneys, feet nerves, and eyes [1,2]. The International Diabetes Federation (IDF) projected an increase in the number of people with diabetes by 51% by the year 2045 [3].

Adherence is defined as the extent to which a patient is committed to follow the healthcare providers’ testaments [4]. Poor adherence has a negative impact on the patient’s health (risk of shortened life span) and economy (increased financial cost and burden) [5]. The American Diabetes Association encourages a reassessment of therapeutic regimen and drug intake on a constant basis every six months at maximum to carry out drug modifications as needed to achieve therapeutic goals [6]. Continuous therapeutic care with good medication adherence is the main aim to help individuals with diabetes to lead a normal life and ultimately reduce occurrence of the long-term complications [7]. Importantly, many factors were found to adversely affect adherence including socio-demographics such as age, financial factors, patient’s education, and social support. Clinical factors as comorbid conditions (mainly cardio-vascular risks) and polypharmacy were also found to affect adherence [8,9].

A patient’s health education, adherence, and concordance have obvious effect in hindering the patient’s clinical improvement [10,11]. For instance, only 50% of individuals with T2DM were highly adherent to their medication during the period of the study [12]. Health education and increasing patient knowledge may also help in increasing adherence to medication in T2DM [13,14]. Good medication adherence in T2DM can also be achieved with long-term use of medication, irrespective of the number of medications used [15]. From our point of view, these factors could help in solving the problem of clinical inertia among healthcare workers in the field of diabetes treatment. In Ethiopia, it was observed that more than half of the participant population with T2DM were not in glycemic control, and this was attributed to non-commitment to prescribed therapy in addition to poor knowledge, and weakness in practicing self-management [16]. While in France, low adherence level to T2DM medications was reported with factors related to the patients themselves, such as age, needing help in taking medications, and other healthcare-related factors. Hereby, better solutions remain a must for better healthcare management [17]. Other factors that may influence adherence may include the age of diabetes, commitment to the eating plan, and poor adherence to medications [18]. Diabetes is a complex disease and more factors were identified in affecting medication adherence. The comorbidity, overall health level, the number of drugs, and complexity of the drug regimen were the main factors. In the authors’ point of view, these factors can lead medical professionals in increasing patients’ adherence [19]. Therefore, encouraging and monitoring adherence in individuals with T2DM may represent an essential and important factor in achieving glycemic control. This can be achieved by health education, monitoring compliance, and even sending mobile text messages [9,20,21]. It was noted that giving individuals with T2DM the choice in making their therapeutic plan may increase the confidence of patients in healthcare providers and can help them to choose the most suitable regimens that achieve their therapeutic goals that suit the patient’s life and decrease clinical inertia [22].

Patient medication counseling is defined as providing medication-related information orally or in writing to the patients or caregivers. The medication counseling should include directions for use, dosage, administration, precautions, storage, and side effects of drugs. Moreover, the counseling could handle non-pharmacological measures such as weight loss, exercise, dietary restrictions, and lifestyle modifications. Pharmacists should counsel individuals with diabetes regarding the importance of medication in managing the disease. This, of course, will help to decrease non-compliance and improve the quality-of-life outcomes in this cohort of individuals [23].

As vital healthcare team members, pharmacists should significantly influence diabetes care and education. They screen patients at high risk for diabetes, set and monitor diabetes treatment goals, and assess the patient’s health status. Pharmacists also train individuals with diabetes on a home glucometer, perform a physical assessment of the patient’s feet, skin, blood pressure, and weight, and assess lipid management, education, and adherence to their medications as well as to the standards of care [24]. The contribution of a clinical pharmacist in diabetes management is essential to the diabetes service, especially in low-resource-setting countries, where there is an extreme shortage in the number of qualified diabetes medical specialists.

Shareef et al. showed the vital role of the pharmacists in increasing patient adherence to treatment through their educational and counseling roles. They concluded that the pharmacist’s role is valuable and well recognized in diabetic patient care [25]. Our study aimed to determine the impact of the clinical pharmacist’s diabetes education on medication adherence in patients with type 2 diabetes mellitus. It is much hoped that this study will contribute in improving the diabetes care services in Sudan. The improvement expected is in decreasing medication errors, enhancing information exchange and collaboration between the diabetes service care providers and the clinical pharmacists. Ultimately, this may provide healthcare authorities with the components of diabetes care, treatment goals, and tools to evaluate the quality of care and encourage physicians and clinical pharmacists to gather data for clinical decision-making and to do their jobs in an evidence-based process.

## 2. Methods

### 2.1. Study Setting

The study was conducted at a diabetes clinic at Omdurman Military Hospital (OMH). The study population was individuals with T2DM attending the diabetes clinic at OMH over 1 year from January 2021 to January 2022.

Study design.

This study was a randomized, double-blind, parallel, superiority controlled trial.

The primary care physicians and the data collectors were blinded.

Trial registration number:

The registration number for this trial is PACTR202311766174946.

### 2.2. Inclusion Criteria

Individuals with type 2 diabetes aged 18 years to 75 years of both sexes and attending the outpatient clinic were included in the study.

### 2.3. Sample Size and Sampling Techniques

Based on the equation used from a website to calculate the sample size in clinical trials (for superiority type trials) [26]:

N1 = {z1 − α/2 × √ p¯ × q¯ × (1 + 1/(k)) + z1 – β × √p1 × q1 + ((p2 × q2)/K)} 2/Δ2

q1 = 1 − p1, q2 = 1 − p2, p¯ = (P1 + kP2)/(1 + K), q¯ = 1 − p¯

where:

p1, p2 = proportion (incidence) of group 1 and group 2 according to a previous study;

Δ = |p2 − p1| = absolute difference between two proportions (0.454–0.303) [27];

n1 = sample size for group #1;

n2 = sample size for group #2;

α = probability of type I error (usually 0.05);

β = probability of type II error (usually 0.2);

z = critical Z value for a given α or β;

K = ratio of sample size for group #2 to group #1;

N1 = {1.96 √ 0.3785 × 0.6215 × (1 + 1/(1)) +0.84 × √0.454 × 0.546 + ((0.303 × 0.697)/1)} 2/(0.151)2;

N1 = 161, N2 = K × N1 = 161 (considered for one arm).

The total sample size was 322, and the study started with 364 participants accounting for any dropouts.

The participants were selected randomly by a simple random sampling method. The total numbers assigned per day were randomly divided into 2 equal groups: one of them was the interventional and the other was the control group.

#### 2.3.1. Intervention

Patients in the control group were managed by their assigned primary care physicians (PCPs) per usual care. Patients in the interventional group were managed by their assigned PCPs per usual care plus pharmacist-managed diabetes clinic (PMDC) visits. During these visits, twelve educational videos about diabetes were provided to the interventional group by the principal investigator. The educational videos were produced by a very experienced clinical pharmacist and covered all information about diabetes medications (mode, time of use, possible contraindications, possible side effects, lifestyle changes, and the importance of patients’ adherence to their healthy diet, medications, and physician’s instructions). The educational videos was produced in a simple Arabic language that is understandable to all patients. The intervention was conducted in the first five months of the study. The PMDC visits were scheduled more frequently during the early months of the interventional period to ensure patient engagement, addressing all patient inquiries about the information mentioned in the videos and providing enough opportunities and time to address all of the patients’ goals and concerns about their disease. We provided three videos per month in the first two months, and the others in the remaining months. The visits to the PMDC took about 15–20 min for each patient. The length of each educational video was about 5 min on average.

#### 2.3.2. Study Outcomes

The main outcome was medication adherence while the secondary outcome was the HbA1c level which was measured at baseline and at 12 months of the study.

### 2.4. Data Collection Tool

We used a pre-structured standardized questionnaire to assess each patient’s adherence to diabetes medications by using the Medication Adherence Rating Scale at baseline and at the end of the study (after 12 months) for the interventional and control group (MARS) [28]. The questionnaire was filled by the data collectors after directly questioning the patients to ensure that all patients understood the questions at the same level. It was filled in at baseline and at the end of the study. Adherence questions consisted of 10 questions with responses of “yes and no”, with the response which indicated better adherence either yes or no, was given “1”, while the response which indicated non-adherence, either yes or no, was given “0”, hence the total scores of attitude ranged from “0 to 10”, with the higher scores indicating better adherence [28]. The reliability analysis of the MARS using Cronbach’s alpha is 0.75.

### 2.5. Statistical Analysis

Data were analyzed by using the Statistical Package for the Social Sciences (SPSS) (version 28) and Microsoft Excel (version 13). Descriptive statistics (frequency tables, means, standard deviations, medians, and IQR) were conducted for describing both the normally distributed and non-normally distributed data. A paired sample T-test was performed to determine any significant differences in the outcome variables between the interventional and control groups from baseline to the end of the study. A statistical test was used at 0.05 alpha level.

## 3. Results

### 3.1. Socio-Demographic Characteristics of the Participants

The sample size in this study was 364 participants; 182 participants were in the interventional group while 182 participants were in the control group. Most of the participants 76.4% (*n* = 278) were females. The mean age of the interventional group was 54.5 (±10) years, while the mean age of the control group was 56 (±9.8) years. Table 1 depicts the rest of the socio-demographic participants’ characteristics.

### 3.2. Disease Characteristics

The median (IQR) duration since DM diagnosis for the total participants was 8 (4–14) years; 42.3% (*n* = 77) of the interventional group were prescribed insulin; of them, 67.5% (*n* = 52) were taking mixed insulin. Nearly the same percentage, 45.6% (*n* = 83) of the control group, were using insulin; of them, 65% (*n* = 54) were taking mixed insulin. Metformin is the most used therapy among the participants; 78% (*n* = 142) of the interventional group and 81.3% (*n* = 148) of control group participants were on metformin, while only 47.8% (*n* = 87) of interventional and 50% (*n* = 91) of control group participants were on sulphonylurea (Table 2).

### 3.3. Comorbidities and Medications Prescribed for the Participants

Hypertension was the major associated co-morbidity. Almost 40% of participants were hypertensive: 39% of the interventional group and 40.1% of the control group. Statins were prescribed for 47.3% of the interventional group and 53.3% of the control group. Calcium channel blockers (CCBs) were the main antihypertensive medications prescribed for both groups. Furthermore, of the participants who had hypertension (*n* = 144), 19.7% (*n* = 14) were taking ACEIs + CCBs; of them, 71.4% (*n* = 10) of the participants were in the interventional group and 29.6% (*n* = 4) of the participants were in the control group; 21.1% (*n* = 15) were taking ARBs+ CCBs; of them, 46.6% (*n* = 7) were in the interventional group and 54.4% (*n*= 8) were in the control group (Table 3).

### 3.4. Treatment Adherence

At baseline, 90.7% (*n* = 165) of the interventional group and 93.4% (*n* = 170) of the control group thought that they could prevent getting sick by staying on medications; 70.9% (*n* = 129) of the interventional group and 68% (*n* = 125) of the control group reported that they forget to take their medications while 62.1% (*n* = 113) of the interventional group and 69.8% (*n* = 127) of the control group reported that they are careless about taking their medication (Table 4).

As Table 5 shows, among the control group, the mean adherence score was 6.8 (±1.6) at baseline and it was 6.7 (±1.6) at the end of the study (*p* < 0.001), while in the interventional group, the adherence score was 6.8 (±1.7) at baseline and it was 7.4 (±1.5) at the end of the study (*p* < 0.001). Independent sample T test results revealed there are statistically significant differences between the mean change in adherence score from baseline to 12 months among the control and interventional groups.

Also, as Table 5 shows, at baseline, the mean HbA1c for the interventional group was 8.7 (±2.2) %, and it was 8.5 (±2.2) % for the control group (*p* = 0.973), while at the end of the study, the mean HbA1c for the interventional group was 6.8 (±0.8) %, and it was 7.7 (±2) % for the control group (*p* = 0.028).

## 4. Discussion

This study was the first interventional study in Khartoum, Sudan evaluated the impact of clinical pharmacist-led diabetes education on medication adherence in Sudanese individuals with type 2 diabetes. In summary, the trial showed significant difference in medication adherence between the interventional and control groups at the end of the study. HbA1c levels improved significantly at the end of the study. Importantly, there were no baseline statistical differences in patients’ socio-demographics and comorbidities. We also noted no significant difference in the prescribed medications between the interventional and control group (*p* > 0.05) as we took into consideration that the two groups should be homogenous in their characteristics.

At baseline, 42.3% of the interventional group were on insulin therapy; of them, 67.5% were taking mixed insulin and 18% were taking glargine insulin. Nearly the same percentage (45.6%) of control participants were on insulin; of them, 65% were taking mixed insulin and 16.9% were taking glargine insulin. At the end of the study, the percentage of insulin users among the interventional group was 45.3%; of them, 61.7% were taking mixed insulin and 27.25% were taking glargine insulin and 46.9% of the control participants were on insulin; of them, 56.6% were taking mixed insulin and 26.5% of the control were taking glargine insulin. It is worth mentioning that the main reason behind the increasing number of glargine insulin users as the study progressed might be due to replacing mixed insulin with the glargine insulin, either by the physician for the control group or by the clinical pharmacist for the interventional group. It was the patient’s preference for the glargine insulin because it has a low incidence of hypoglycemic incidence and it is a long-acting insulin that is administered once daily when compared with the mixed insulin, enabling the patients to be more adhered to their medication for regular use.

The monitoring of patient adherence should not be restricted to medication therapy; it should also include blood glucose monitoring, dietary restrictions, and lifestyle recommendations. Clinical pharmacists assess adherence by patient interview and review of prescription-filling practices. They also promote patient adherence to medications by advising them to use tools such as simplified therapy dosing schedules and minimization of unnecessary therapies [29]. One of the best ways to improve medication adherence is patient medication counseling.

A patient’s health education, adherence, and concordance have an obvious effect in hindering a patient’s clinical improvement [10,11]. Poor adherence to anti-diabetic medications is a common problem among individuals with diabetes. These two facts warrant effective intervention to improve adherence. Unfortunately, no effective solution yet found the best way to improve adherence to medication [30,31,32]. In our study, the mean adherence score was increased by 0.6 from baseline to the end of the study for the interventional group, while for the control group, it was decreased by 0.1 (*p* < 0.001). Therefore, the current study clearly showed that intervention by a clinical pharmacist showed significant improvement in adherence to the anti-diabetic medication.

Some studies conducted by clinical pharmacists to evaluate the impact of a pharmacist’s education on medication adherence using the Morisky scale as a tool for adherence assessment found that the adherence in the interventional group at the end of the study was significantly improved compared to the control group (*p* < 0.01) [33,34]. In addition, Lee et al. also showed that medication adherence assessed by the Morisky scale was improved after an educational program conducted by clinical pharmacists [35]. Our study highlighted the impact of the educational program conducted by the clinical pharmacist for the interventional group. The current study is not without limitations. First, the study was conducted at one hospital-based diabetes clinic which can limit the generalizability of the results. Second, the estimation of the medication adherence rate in our study was based on patient self-reporting which may lead to overestimation of the parameters. Using a combined approach of self-reported and observational methods would be more beneficial, but as stated by a recent literature search, standardized self-report scale measures can achieve the objectives adequately.

## 5. Conclusions

Adherence among the intervention group was increased significantly from baseline to the end of the study when compared to the control group and this might be due to the educational program provided by the clinical pharmacist. Patient education and counseling by the clinical pharmacists in order to improve medication adherence will further strengthen the significant role of clinical pharmacists in the management of diabetes mellitus.

## Figures and Tables

**Table 1:** Socio-demographic characteristics of the participants (*n* = 364).

	Responses	Study Group				Total	
		Intervention (*n* = 182)		Control (*n* = 182)		*n* = 364	
		*n*	%	*n*	%	*n*	%
Gender	Males	35	19.2	51	28	86	23.6
	Females	147	80.8	131	72	278	76.4
Age	Mean (±SD)	54.5 (±10)		56 (±9.8)		55.2 (±9.9)	
Age category	<40 years	18	9.9	16	8.8	34	9.3
	41–50 years	59	32.4	42	23.1	101	27.7
	51–60 years	57	31.3	65	35.7	122	33.5
	61–70 years	38	20.9	49	26.9	87	23.9
	>70 years	10	5.5	10	5.5	20	5.5
Marital status	Married	149	81.9	158	86.8	307	84.3
	Unmarried	5	2.7	3	1.6	8	2.2
	Divorced	7	3.8	2	1.1	9	2.5
	Widowed	21	11.5	19	10.4	40	11.0
Residence	Urban	165	90.7	150	82.4	315	86.5
	Rural	17	9.3	32	17.6	49	13.5
Educational level	Illiterate	26	14.3	37	20.3	63	17.3
	Primary	51	28.0	68	37.4	119	32.7
	Secondary	73	40.1	55	30.2	128	35.2
	University graduate	29	15.9	22	12	51	14.0
	Postgraduate	3	1.6	00	00	3	0.8

SD: standard deviation. Note: test was used at alpha level 0.05.

**Table 2:** Hypoglycemic agents that were prescribed for the participants (*n* = 364).

Variable	Responses	Study Group				Total	
		Intervention (*n* = 182)		Control (*n* = 182)		*n* = 364	
		*n*	%	*n*	%	*n*	%
Duration of diabetes mellitus	Median (IQR)	7 (4–14)		8 (4–14)		8 (4–14)	
Sulphonylurea	-	87	47.8	91	50.0	178	48.9
Duration of sulphonylurea/years	Median (IQR)	5 (1–7)		5.5 (2–12.3)		5 (2–9)	
Duration of sulphonylurea	<1 year	15	17.2	14	15.4	29	16.4
	1–5 years	38	43.7	31	34.1	69	39.0
	6–10 years	24	27.5	19	20.9	43	24.3
	11–15 years	6	6.9	17	18.7	23	13.0
	>15 years	4	4.5	9	9.9	13	7.3
Metformin	-	142	78.0	148	81.3	289	79.4
Duration of metformin/years	Median (IQR)	5.5 (2–10)		6.5 (3–13)		6 (3–11)	
Duration of metformin	<a year	14	9.9	12	8.1	26	9.0
	1–5 years	57	40.1	52	35.1	109	37.6
	6–10 years	43	30.3	34	23.0	77	26.6
	11–15 years	19	13.4	32	21.6	51	17.6
	>15 years	9	6.3	18	12.2	27	9.3
Insulin	Yes	77	42.3	83	45.6	164	45.1
Duration/years	<a year	15	19.5	15	18	30	18.3
	1–5 years	31	40.3	40	48.2	71	43.2
	6–10 years	16	20.8	16	19.3	32	19.5
	11–15 years	6	7.8	6	7.2	12	7.3
	>15 years	4	5.2	4	4.8	8	4.9
Type of insulin used	Soluble	2	2.6	3	3.6	5	3.1
	Mixed	52	67.5	54	65	106	64.6
	Glargine + Soluble	7	9	12	14.5	19	11.6
	Glargine	14	18	14	16.9	28	17
	Soluble + Mixed	2	2.6	00	00	2	1.2
Vildagliptin	-	2	1.1	3	1.6	5	1.4
Vildagliptin + metformin	-	3	1.6	1	0.5	4	1.1

IQR: Interquartile range. Note: test was used at alpha level 0.05.

**Table 3:** Co-morbidities and the medications prescribed for the participants (*n* = 364).

Variable	Responses	Study Group				Total	
		Intervention (*n* = 182)		Control (*n* = 182)		*n*	
		*n*	%	*n*	%	*n*	%
Hypertension	-	71	39.0	73	40.1	144	39.6
Duration of hypertension	Median (IQR)	8 (3–12)		10 (3–15)		8 (3–14)	
Antihypertensive medications	CCB	43	53.7	40	47	83	50.3
	ARBS	21	26.3	15	17.6	36	21.8
	ACEIs	11	13.8	21	24.7	32	19.4
	ARBs + thiazides	4	5	2	2.4	6	3.7
	Others	1	1.2	7	8.2	8	4.8
Dyslipidemia medications	Statins	86	47.3	97	53.3	183	50.2
	Fibrates	1	0.5	3	1.6	4	1.1

IQR: Interquartile range, CCB: calcium channel blockers, ARBs: angiotensin receptor blockers, ACEIs: angiotensin-converting enzyme inhibitors. Note: test was used at alpha level 0.05.

**Table 4:** Participants’ treatment adherence among the studied population at baseline (*n* = 364).

Adherence Items	Study Group			
	Intervention (*n* = 182)		Control (*n* = 182)	
	Yes	No	Yes	No
1. Do you ever forget to take your medication?	129 (70.9)	53 (29.1)	125 (68)	57 (31.1)
2. Are you careless at times about taking your medication?	113 (62.1)	69 (37.9)	127 (69.8)	55 (30.2)
3. When you feel better, do you sometimes stop taking your medicine?	74 (40.7)	108 (59.3)	82 (45.1)	100 (54.9)
4. Sometimes if you feel worse when you take the medicine, do you stop taking it?	102 (56)	80 (44)	103 (56.6)	79 (43.4)
5. I take my medication only when I am sick.	48 (26.4)	134 (73.6)	34 (18.7)	148 (81.3)
6. It is unnatural for my mind and body to be controlled by medication.	19 (10.4)	163 (89.6)	19 (10.4)	163 (89.6)
7. My thoughts are clearer on medication.	163 (89.6)	19 (10.4)	166 (91.2)	16 (8.8)
8. By staying on medication, I can prevent getting sick.	165 (90.7)	17 (9.3)	170 (93.4)	12 (6.6)
9. I feel weird, like a ‘zombie’, on medication.	18 (9.9)	164 (90.1)	18 (9.9)	164 (90.1)
10. Medication makes me feel tired and sluggish.	39 (21.4)	143 (78.6)	41 (22.5)	141 (77.5)

**Table 5:** Comparison of adherence scores and HbA1c in both groups at baseline and follow-up at 12 months.

Variables	Intervention		Control		*p* Value Baseline	*p* Value 12 Months
	Baseline	12 Months	Baseline	12 Months		
Adherence score	6.8 (±1.7)	7.4 (±1.5)	6.8 (±1.6)	6.7 (±1.6)	0.975	0.000
HbA1c	8.7 (±2.2)	6.8 (±0.8)	8.5 (±2.2)	7.7 (±2)	0.973	0.028

## Data Availability

Data are contained within the article.
